# Perforating duodenal ulcer with umbilical herniation as a metastatic complication of primary signet ring cell carcinoma of the breast

**DOI:** 10.1093/jscr/rjab034

**Published:** 2021-03-10

**Authors:** Daniel R Principe, Andreea Raicu, Jose Cataneo, Holly R Beverley, Matthew Hyser

**Affiliations:** Medical Scientist Training Program, University of Illinois College of Medicine, Chicago, IL, USA; Department of Surgery, University of Illinois College of Medicine, Chicago, IL, USA; University of Illinois, Metropolitan Group Hospitals General Surgery Residency, Advocate Illinois Masonic Hospital, Chicago, IL, USA; Department of Surgery, University of Illinois College of Medicine, Chicago, IL, USA; University of Illinois, Metropolitan Group Hospitals General Surgery Residency, Advocate Illinois Masonic Hospital, Chicago, IL, USA; Library of the Health Sciences, University of Illinois College of Medicine, Chicago, IL, USA; Department of Surgery, AMITA St. Francis Hospital, Evanston, IL, USA

## Abstract

Primary signet ring cell carcinoma (SRCC) of the breast is extremely rare, and the associated patterns of metastatic dissemination poorly described. Here, we report the case of a 61-year-old woman presenting with acute abdominal pain. Esophagogastroduodenoscopy revealed a non-bleeding erosive gastropathy, which was biopsied and found significant for a poorly differentiated, GATA3-positive SRCC. The patient was lost to follow up until re-presenting 6 months later with a perforating duodenal ulcer and umbilical herniation. Biopsies of umbilical hernia sack contents were significant for an estrogen receptor (ER) positive SRCC, and breast examination identified a right breast mass significant for an ER positive lobular carcinoma with signet ring features, thereby affirming the diagnosis of metastatic SRCC of the breast. This case offers insight into an advanced form of a rare clinical entity, and suggests that staining for breast markers such as GATA3 should be considered for all biopsies significant for SRCC.

## INTRODUCTION

Breast cancer is the most frequently diagnosed malignancy in the USA, affecting one in eight women over the course of their lifetime [1]. The clinical paradigm for breast cancer treatment is dictated largely by surgical candidacy and molecular subtype, the latter based predominantly on the expression or absence of clinically actionable receptors. Within these subtypes, there are several less common breast cancer histotypes that are also recognized, many of which may also have their own unique implications regarding treatment plan and prognosis.

Of these subvariants, primary signet ring cell carcinoma (SRCC) of the breast is among the rarest and least understood. Signet ring breast cancers have poorer outcomes than those lacking signet ring features, including an increased incidence of axillary lymph node metastases [2, 3]. Though primary SRCC has been recognized as a bona fide breast cancer subtype since 2003, given the small number of reported cases, the associated patterns of metastatic dissemination are poorly described.

## CASE PRESENTATION

**Figure 1:**
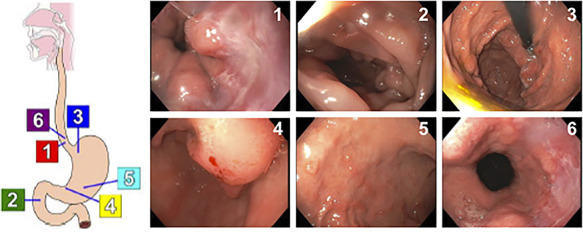
Results of EGD showing dispersed, 3-mm non-bleeding erosions in the gastric antrum.

**Figure 2:**
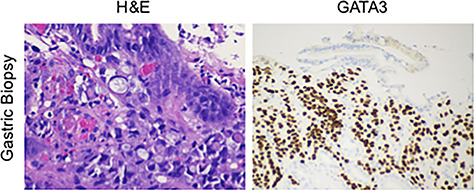
Tissues from the gastric biopsy were stained with either H&E or for GATA3, showing a poorly differentiated signet ring carcinoma of breast origin.

A 61-year-old woman presented to the emergency department with acute onset abdominal pain, nausea and non-bilious emesis. Her medical history was significant for rheumatoid arthritis, hypertension, chronic kidney disease and long-term heroin abuse. Physical examination was significant for mild epigastric tenderness with no peritoneal signs, and lab work was unremarkable at this time. Given her symptoms and extensive medical history, the patient underwent an esophagogastroduodenoscopy (EGD). The distal esophagus appeared red and macerated as a result of recent vomiting, and we observed dispersed, multiple 3-mm non-bleeding erosions in the gastric antrum with no stigmata of recent bleeding (Fig. 1). Gastric biopsies revealed a poorly differentiated, GATA3 positive, CDX2 negative signet ring carcinoma (Fig. 2). The patient was referred to medical oncology, but was lost to followup.

**Figure 3:**
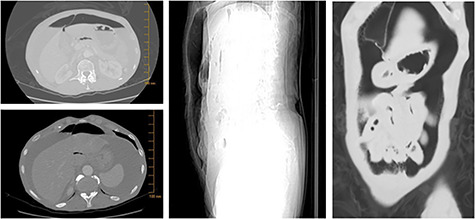
Imaging studies revealing free air in the abdomen.

**Figure 4:**
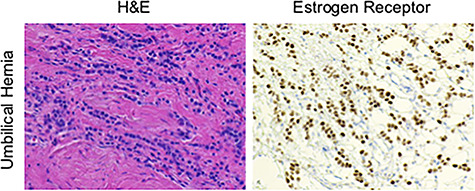
Contents of the umbilical hernia were stained with either H&E or for the ER, showing a poorly differentiated, ER positive, signet ring carcinoma with lobular features.

Six months later, she re-presented to the emergency department with acute onset abdominal pain, nausea and non-billous emesis. Physical examination was again significant for mild epigastric tenderness with no peritoneal signs, however imaging studies revealed free air in the abdomen (Fig. 3). The patient was diagnosed with a perforated viscus, and taken for urgent surgery. She was found to have a 4-mm duodenal perforation, which was excised and repaired with a Graham patch. Laparotomy was also significant for an umbilical hernia with fat, which was repaired primarily and contents sent to pathology for evaluation. While the hernia contents appeared grossly normal on examination, biopsy was found significant for a poorly differentiated, GATA3 and estrogen receptor (ER) positive, CDX2 negative SRCC with lobular features (Fig. 4).

**Figure 5:**
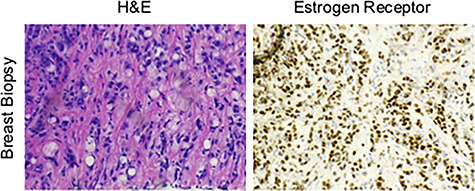
Tissues from breast biopsy were stained with either H&E or for the ER, showing an invasive lobular carcinoma with signet ring cell features.

On subsequent breast examination, a small mass was identified in the right breast. This was biopsied and sent to pathology where it was also found significant for invasive lobular carcinoma with signet ring cell features (Fig. 5). The patient was diagnosed with primary SRCC of the breast and referred to medical oncology for evaluation. However, the patient died prior to her first appointment of complications related to chronic drug abuse.

## DISCUSSION

Here we present the highly unusual case of a patient with primary breast SRCC that initially presented as a metastatic lesion to the gastric antrum, and re-presented as a perforating duodenal ulcer and umbilical herniation. Primary breast SRCC is among the most rare and least understood forms of breast cancer. A recent study of 3587 breast cancer patients identified only 25 cases of SRCC (0.7%). Of these 25 cases, most were ER+ and HER2−, as was our patient. Additionally, 19 of 25 (76%) had a mixed histology, only 6 (24%) of which were SRCC with invasive lobular carcinoma as seen with our patient [4]. Thus, per this study’s estimation our patient’s histotype represents only 0.17% of all breast cancer diagnoses.

As primary breast SRCC is so rare, there are few studies describing its patterns of metastatic dissemination, particularly for cases co-mixed with invasive lobular carcinoma. The aforementioned study has provided some insight into primary breast SRCC metastasis, as 7 of 25 (28%) primary breast SRCC patients developed distant metastases, with secondary lesions observed in the bone, liver and ovaries [4]. The ovary appears to be the most frequently reported site of primary breast SRCC metastasis, though two additional cases of gastric metastases from primary breast SRCC have been reported [5–7]. Interestingly, ~90% of all SRCC tumors originate in the stomach, and in very rare instances, primary gastric SRCC can metastasize to the breast [8]. As the treatment for breast and gastric SRCCs can differ significantly, it is imperative to determine the tissue of origin for any lesion with signet ring cell features. Thus, we recommend that all gastric biopsies significant for SRCC be subjected to staining for breast lineage markers.

Although several stains can provide insight into a potential breast origin, GATA3 may be more useful in this application than other breast markers, particularly when evaluating gastric biopsies. While GATA3 is an established breast lineage marker present in 96% of breast cancer metastases [9], GATA3 is not generally expressed in primary gastric SRCC [10]. Hence, GATA3 has been suggested as a reliable means of differentiating between primary gastric and metastatic breast SRCC lesions involving the stomach. Conversely, CDX2 is expressed in most gastric SRCCs, yet is rarely expressed in primary breast SRCC [10]. Hence, CDX2 may also be informative in differentiating between SRCC of breast and gastric origin.

Additionally, this case serves as a reminder of the importance of continued follow up for patients with substance abuse disorders. Though extensive attempts were made to contact the patient following the initial consult, none were successful and despite our efforts she was lost to follow up. In the time that we were unable to contact our patient, she developed extensive new metastases requiring additional surgery and re-hospitalization. This raises the question as to whether her disease progression could have been avoided should she have followed up with medical oncology, and is a tragic demonstration of how substance abuse disorders can cooperate with additional health issues. Therefore, care providers must consider providing adequate addiction-counseling resources an equal priority to treating a comorbid malignancy in order to prevent such outcomes as our patient.

## CONFLICT OF INTEREST STATEMENT

The authors have no potential conflicts to disclose.

## FUNDING

This work was supported by the National Cancer Institute of the National Institutes of Health under Award Number F30CA236031 to D.R. Principe.
